# Ethephon As a Potential Abscission Agent for Table Grapes: Effects on Pre-Harvest Abscission, Fruit Quality, and Residue

**DOI:** 10.3389/fpls.2016.00620

**Published:** 2016-05-30

**Authors:** Giuseppe Ferrara, Andrea Mazzeo, Angela M. S. Matarrese, Carmela Pacucci, Antonio Trani, Matthew W. Fidelibus, Giuseppe Gambacorta

**Affiliations:** ^1^Dipartimento di Scienze del Suolo, della Pianta e degli Alimenti, University of Bari ‘Aldo Moro’Bari, Italy; ^2^Department of Viticulture and Enology, University of California, DavisDavis, CA, USA

**Keywords:** ethephon, table grape, thompson seedless, crimson seedless, fresh-cut

## Abstract

Some plant growth regulators, including ethephon, can stimulate abscission of mature grape berries. The stimulation of grape berry abscission reduces fruit detachment force (FDF) and promotes the development of a dry stem scar, both of which could facilitate the production of high quality stemless fresh-cut table grapes. The objective of this research was to determine how two potential abscission treatments, 1445 and 2890 mg/L ethephon, affected FDF, pre-harvest abscission, fruit quality, and ethephon residue of Thompson Seedless and Crimson Seedless grapes. Both ethephon treatments strongly induced abscission of Thompson Seedless berries causing >90% pre-harvest abscission. Lower ethephon rates, a shorter post-harvest interval, or berry retention systems such as nets, would be needed to prevent excessive pre-harvest losses. The treatments also slightly affected Thompson Seedless berry skin color, with treated fruit being darker, less uniform in color, and with a more yellow hue than non-treated fruit. Ethephon residues on Thompson Seedless grapes treated with the lower concentration of ethephon were below legal limits at harvest. Ethephon treatments also promoted abscission of Crimson Seedless berries, but pre-harvest abscission was much lower (≅49%) in Crimson Seedless compared to Thompson Seedless. Treated fruits were slightly darker than non-treated fruits, but ethephon did not affect SSC, acidity, or firmness of Crimson Seedless, and ethephon residues were below legal limits.

## Introduction

Table grapes that meet minimum maturity standards, including sugar and acid content, and the ratio of sugar to acid, are harvested by hand and typically marketed as entire or partial clusters. The quality and value of the grapes are strongly affected by the size, texture, and color of the individual berries, and the overall appearance of the cluster (Fidelibus et al., 2010). These quality attributes are commonly achieved, in part, through the use various plant growth regulators (PGRs), agrochemicals with plant hormones or hormone-like compounds as active ingredients (Ferrara et al., 2013, 2015). For example, gibberellic acid is used to thin and size berries, forchlorfenuron is used to increase berry size, and firmness (Ferrara et al., 2014), and ethephon (2-chloroethylphosphate acid) and abscisic acid may also be used to improve the color of red grapes (Fidelibus et al., 2010; Ferrara et al., 2013, 2015).

Though the vast majority of table grapes are sold as entire or partial clusters, there is growing interest in marketing stemless fresh-cut grapes (Kou et al., 2007). However, destemming may damage grape berries, stimulating decay, and diminishing quality (Kou et al., 2006, 2007). Mechanical damage associated with destemming might be minimized through the use of abscission agents, PGRs which reduce fruit detachment force (FDF) and promote the development of a dry stem scar, an abscission layer between the berry and pedicel (Fidelibus et al., 2007; Ferrara et al., 2010). Research on the potential use of abscission agents as mechanical harvest aids for wine or raisin grapes have shown that 1-aminocyclopropane-1-carboxylic acid (ACC), coronatine, ethephon, and methyl jasmonate (MeJA) stimulate abscission of mature grape berries (Hedberg and Goodwin, 1980; Szyjewicz et al., 1984; Fidelibus et al., 2007; Uzquiza et al., 2013, 2014). Of those, ethephon is the only compound registered for use on grapes, though the registrations are for improving the color of red and black fruited grapes, or hastening grape maturity, both at considerably lower use rates than what is required to stimulate berry abscission.

Ethephon is an ethylene-releasing molecule. Stable in a low pH solution, it hydrolyses in the higher pH of plant tissues releasing ethylene, a gaseous plant growth regulator (Royer et al., 2006). Ethephon's chemical characteristics enable growers to apply it to grapes and other plants in the field with commercial spray equipment, and thereby stimulate ethylene-dependent reactions. Ethephon absorption by plant tissues is influenced by temperature, relative humidity, and pH of the surface on which the spray droplets are deposited (Turnbull et al., 1999). Hedberg and Goodwin (1980) suggested that ethephon absorption by plant tissues is predominantly cuticular rather than stomatal and Nir and Lavee (1981) found that the thickness and composition of cuticle layers play an important role in penetration. How the molecule diffuses within the plant is not yet well understood. Studies conducted with the 2-chloroethylphosphoric acid marked with the ^14^C showed limited and mainly basipetal mobility (Weaver et al., 1972).

Ethylene regulates many aspects of fruit development including maturation, senescence, and abscission (Szyjewicz et al., 1984). Grape is considered non climacteric but an ethylene peak detected at veraison, the onset of ripening, may be higher than the physiological threshold for metabolic activities (Abeles et al., 1992), and Giovannoni (2001) reported some aspects of non-climacteric ripening are probably associated with ethylene responses. Likewise, Chervin et al. (2004) reported that ethylene seems required for the increase in berry diameter, decrease in berry acidity and anthocyanins accumulation that occurs after veraison. Regardless of the endogenous role of ethylene in grape berry development, ethephon has well-established commercial uses in viticulture to promote fruit maturation-related processes, including the synthesis and accumulation of anthocyanins in berries and the accumulation of soluble solids (Szyjewicz et al., 1984; Shulman et al., 1985), and grape berries, which generally don't abscise naturally, can be induced to abscise with exogenous application of ethephon or other compounds that stimulate ethylene production by grape berries (Uzquiza et al., 2014).

The potential for ethephon as an abscission agent for table grapes is a relatively new concept that has been little studied (Fidelibus et al., 2007; Ferrara et al., 2010). If ethephon is to ever be registered for that use, the potential for excessive residues will have to be considered. This is especially important since relatively high rates of ethephon are needed to stimulate grape berry abscission, the process occurs quickly (so post-harvest interval may be short), and berries are consumed whole, without peeling. Therefore, the present study aimed to verify the effects of ethephon on the abscission of grape berries of two globally important seedless table grape *Vitis vinifera* cultivars, and on the residual concentration of ethephon in the berry in order to evaluate its potential for aiding in the production of fresh-cut fruit.

## Materials and methods

### Experimental site

Experiments were carried out in 2012 in Thompson Seedless and Crimson Seedless table grape vineyards located in the countryside of Adelfia (Bari) and Francavilla Fontana (Brindisi), respectively. Both Thompson Seedless and Crimson Seedless were grafted onto 140 Ru (*Vitis berlandieri* × *V. rupestris*) and trained to an overhead trellis system (tendone), with the first spaced 2.8 m between rows and 2.5 m within rows and the latter 3.0 m between rows and 2.5 m within rows. Grapevines were cane pruned (four canes per vine) with 12–14 nodes per cane. Vines were drip irrigated from May to September (1800–2000 m^3^/ha). A single irrigation pipeline was positioned on the soil with three drippers for each vine (4 L/h). Soil water potential was kept below −300 kPa, and leaf water potentials were maintained at values < −0.6 MPa. Fertilizer addition, pest control, and other vineyard operations (gibberellic acid application, berry thinning, leaf removal, and lateral shoot thinning) were conducted according to local practices.

A randomized block design was used with three blocks and three treatments, and each treatment in the block consisted of six grapevines selected with a uniform number of clusters. Each treatment consisted of: (1) control, (2) ethephon at 1445 mg/L, (3) ethephon at 2890 mg/L. The concentrations used in this trial were established on results obtained in preliminary studies (Ferrara, unpublished data). Ethephon was dispersed in water with 0.1% (v/v) of a surfactant (Astrol nuovo, Dow AgroSciences, Bologna, Italy) and applied directly to the clusters of vines selected for abscission treatments. Clusters from control vines were treated with water containing the surfactant only. The ethephon or control solutions were applied with a handheld sprayer until run-off when the fruits reached sufficient soluble solids (at least 16° Brix) for harvest (9 and 17 September for Thompson Seedless and Crimson Seedless, respectively). After the berries dried, each cluster was enclosed in a mesh bag to collect any berries that may abscise.

### Physical and chemical analyses

Berries were sampled before treatment, 2 h after treatment and in successive days, as reported in Table 1. Measurements of FDF, berry skin color, and firmness were as described previously (Ferrara et al., 2013). In brief, FDF was determined as the force required to detach the berry from the rachis as measured with a mechanical gauge (PCE Italia s.r.l., Capannori, Italy). Berry skin color was measured with a chroma meter (CR-400, Konica Minolta, Tokyo, Japan) that reports color in terms of lightness (L^*^), chroma (C^*^), and hue (h°), where L^*^ refers to the lightness of a color, from black = 0, to white = 100, C^*^ refers to the intensity of a color, with 0 being achromatic, and h° is the position on the color wheel where 0° = red, 90° = yellow, 180° = green, and 270° = blue (McGuire, 1992). Berry firmness was measured with a 2-mm needle digital penetrometer (FM200, PCE Italia s.r.l., Capannori, Italy) in accordance to previously described procedures. A hand-held, temperature compensating digital refractometer and an automatic titrator (PH-Burette 24, Crison Instruments, Barcelona, Spain) were used for the following determinations: (i) soluble solids content (SSC), (ii) pH, (iii) titratable acidity (TA) (as g tartaric acid/L juice at pH 8.1). For all these measurements, 10 clusters from each vine were selected and three berries from each cluster (top, middle, and bottom section) were sampled to measure the FDF and three berries for the other measurements.

**Table 1:** **Sampling dates of the berries**.

**Variety**	**Stage**	**Date**
Thompson seedless	Pre-treatment	9 September
	Post-treatment (2 h)	9 September
	Post-treatment	14 September
	Harvest	21 September
Crimson seedless	Pre-treatment	17 September
	Post-treatment (2 h)	17 September
	Post-treatment	21 September
	Post-treatment	27 September
	Harvest	7 October

*Grapes reached 16% SSC on the 7–8 and 15–16 September for Thompson Seedless and Crimson Seedless, respectively. Clusters were ready for commercial harvest*.

**Figure 1:**
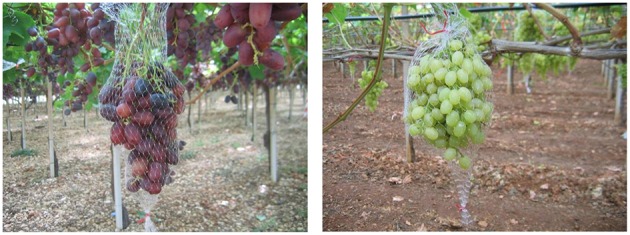
**Mesh bags to prevent pre-harvest berry loss of Thompson Seedless (right) and Crimson Seedless (left) table grapes**.

Pre-harvest abscission was determined by counting any abscised berries that had collected in the mesh bags on observation days (Figure 1). Abscised berries were placed in plastic bags and stored in a portable ice box for transport to the laboratory where the integrity of the berry, including the presence/absence of a pedicel, and a wet or dry stem scar was observed with the aid of a binocular microscope at 30 × (Nikon SMZ800, Japan). Berries that abscised pre-harvest and those that fell during harvest, handling or after light shaking constituted the total percentage of dropped berries. The abscised berry percentage was calculated as [(Berry abscised before harvest + Berry abscised after mild shaking-handling)/(Total weight of cluster) × 100].

### Analyses of ethephon residues in the berry

Ethephon residues were determined according to the method proposed by Takenaka (2002). For each treatment 30 berries were randomly collected from 10 clusters, stored in a portable ice box, and carried to the laboratory for analysis. Cartridges SPE NH_2_ 500 mg of Phenomenex (Torrance, CA, USA) activated as suggested by manufacturer were used in the purification step. The purified samples were evaporated to dryness with a rotavapor at 40°C, taken up with 1 ml of methanol and subjected to derivatization. One hundred microliters of reconstituted samples were transferred to 1.5 mL eppendorf, diluted with 500 μL of acetone and derivatized by adding 10 μL of trimethylsilyldiazomethane (SIGMA-Aldrich). The reaction vials were maintained at 50°C for 30 min, then 10 μL of 1 M acetic acid in methanol were added in order to stop the reaction. After centrifugation, 2 μL of the clear upper phase were injected in the GC-MS system.

### GC-MS analysis

Ethephon was determined using a gas chromatograph 6850 (Agilent) coupled with a single quadrupole mass spectrometer 5975C (Agilent). The gas chromatograph was equipped with a capillary Rtx-CL Pesticides Column 30 m × 0.25 mm and 0.25 μm of film thickness. The operating conditions were:

- Injector temperature 250°C in splitless mode for 60 s, then in split mode (split ratio 1:50),
- Oven 50°C for 2 min, then increased to 250°C at 15°C/min,
- Carrier gas He, constant flow of 1 ml/min,
- Detector temperature150°C, electron impact 70 eV, source, and interface temperature 250°C, acquisition mode in SIM (single ion monitoring) at 110 uma, solvent delay 5 min.

**Figure 2:**
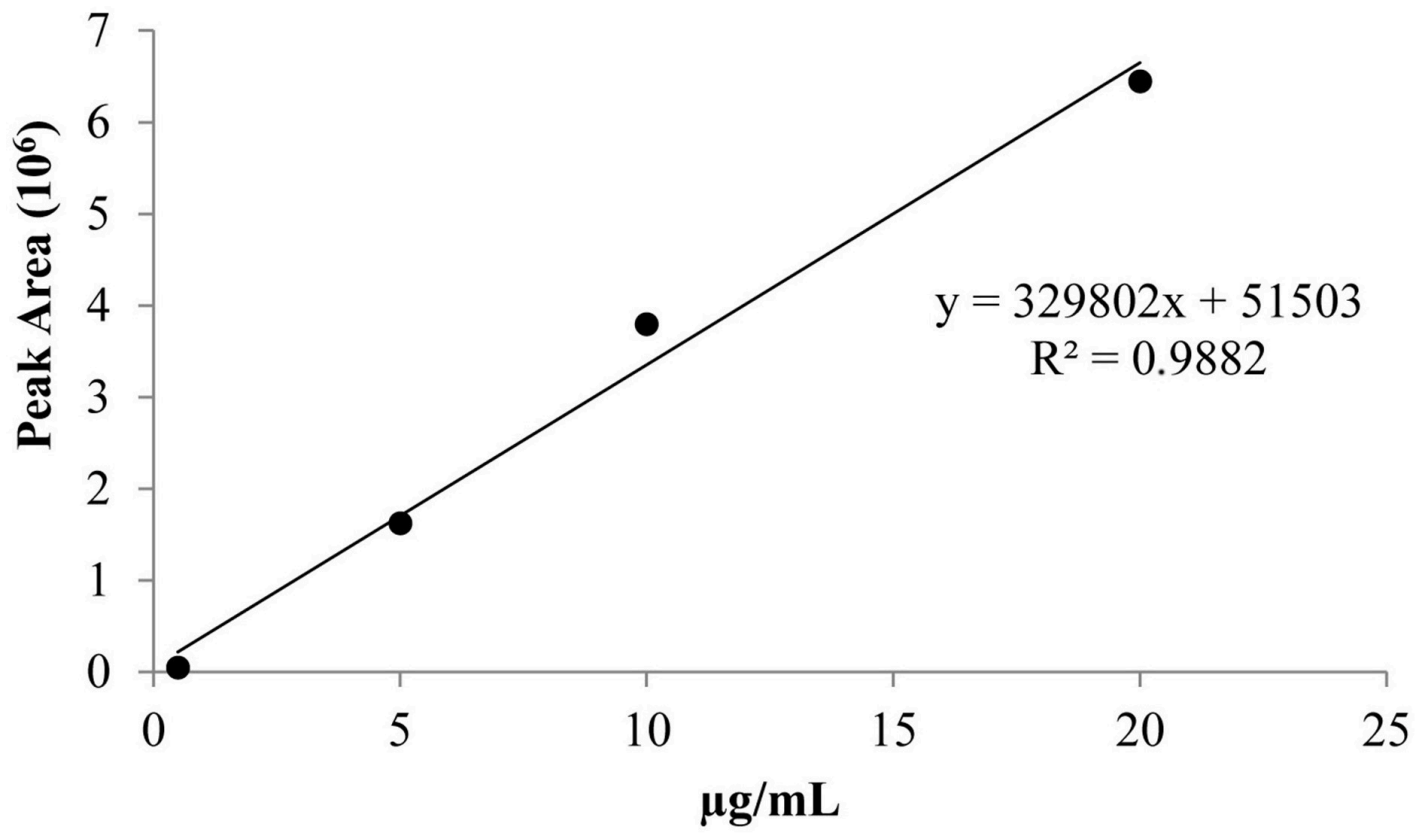
**Calibration curve for ethephon analysis in the berry**.

The derivatized pure ethephon standard was injected under the experimental conditions, acquiring the full mass spectra, and the retention time. The mass spectrum was recognized by NIST library as dimethyl-ethyl phosphate (the methylated form of ethephon) with a level of matching above 70%. The calibration curve (Figure 2) was achieved by injecting in triplicate a pure ethephon standard at four increasing concentrations (0.5, 5, 10, 20 μg/ml), after derivatization with trimethylsilyldiazomethane.

The performance of the calibration was as follows: coefficient of determination (*R*^2^) 0.9882, detection limit 0.06 μg/mL at S/N ratio of 3/1, quantification limit 0.1 μg/mL at S/N ratio >10. These concentrations corresponded to 0.0024 and 0.012 mg of ethephon per kg of fresh grape, clearly below 0.7 mg/kg of product, the maximum allowable residue limits (MRLs) established by European Food Safety Authority (EFSA) (2009) for table grapes.

### Statistical analysis

Analysis of variance (ANOVA) was performed with the software XLSTAT-Pro (Addinsoft, France), the level of significance was set at 0.01. The assumptions of variance were verified with the Levene test (homogeneity of variance) and the Lillefors test (normal distribution). The mean values obtained for the different treatments were statistically separated by using the REGWQ test. Crimson Seedless berries may vary in hue (h°) from red-yellow to red-purple. Such colors result in a range of h° that bracket red, which has a hue of 0. Most h°-values were between 0 and 18° but values of a few measurements were between 315 and 360°, indicating a red-purple berry. Hue angles between 360 and 315° were transformed into negative numbers by subtracting 360, thereby establishing a continuous range of h°-values from which the average h° could be correctly calculated. As regard the analysis of residuals, we used the Kinfit package—Routines for fitting kinetic model to chemical degradation data—in R 3.1.2 to compute DT50 and DT90 values.

## Results and discussion

### Physical and chemical analyses in Thompson seedless

Ethephon application did not affect berry color of Thompson Seedless until 14 days after treatment (Table 2). At that time, ethephon-treated fruit was darker in color (lower L^*^), and had lower C^*^ and a greater h°, indicating the fruit were somewhat more yellow colored than non-treated fruit and generally had a more mature appearance. These findings are consistent with other reports that ethephon affects berry skin color by stimulating the accumulation of phenolic compounds (El-Kereamy et al., 2003; Nikolaou et al., 2003; Lombard et al., 2004; Uzquiza et al., 2015).

**Table 2:** Effects of different ethephon treatments on units of color space (L^*^, C^*^, and h°) of the berry skin, fruit detachment force (FDF), and firmness (whole berry and pulp) of Thompson seedless.

		L^*^	C^*^	**h°**	**FDF^3^**	**Berry^3^**	**Pulp^3^**
	**9 SEPTEMBER**						
Treatments	Control	43.1	16.1	109.2	4.00	4.04	0.70
	Eth 10^1^	43.2	15.6	109.8	3.92	3.99	0.67
	Eth 20^2^	43.7	16.2	109.9	3.79	4.12	0.70
	**21 SEPTEMBER**						
	Control	44.5^A^	15.4^A^	109.9^B^	2.96	3.77	0.38
	Eth 10	41.1^B^	14.1^B^	111.7^A^	2.86	3.75	0.37
	Eth 20	41.5^B^	14.7^AB^	110.3^AB^	2.83	3.05	0.36
Time	**DATES**						
	9 Sept.	43.3^A^	15.9^A^	109.6^B^	3.91^A^	4.05^A^	0.69^A^
	21 Sept.	42.4^B^	14.7^B^	110.7^A^	2.88^B^	3.52^B^	0.37^B^

*Means followed by different letters in each column are significantly different (REGWQ, P ≤ 0.01).^1^Eth 10, ethephon at a concentration of 1445 mg/L; ^2^Eth 20, ethephon at a concentration of 2890 mg/L; ^3^values in N*.

**Figure 3:**
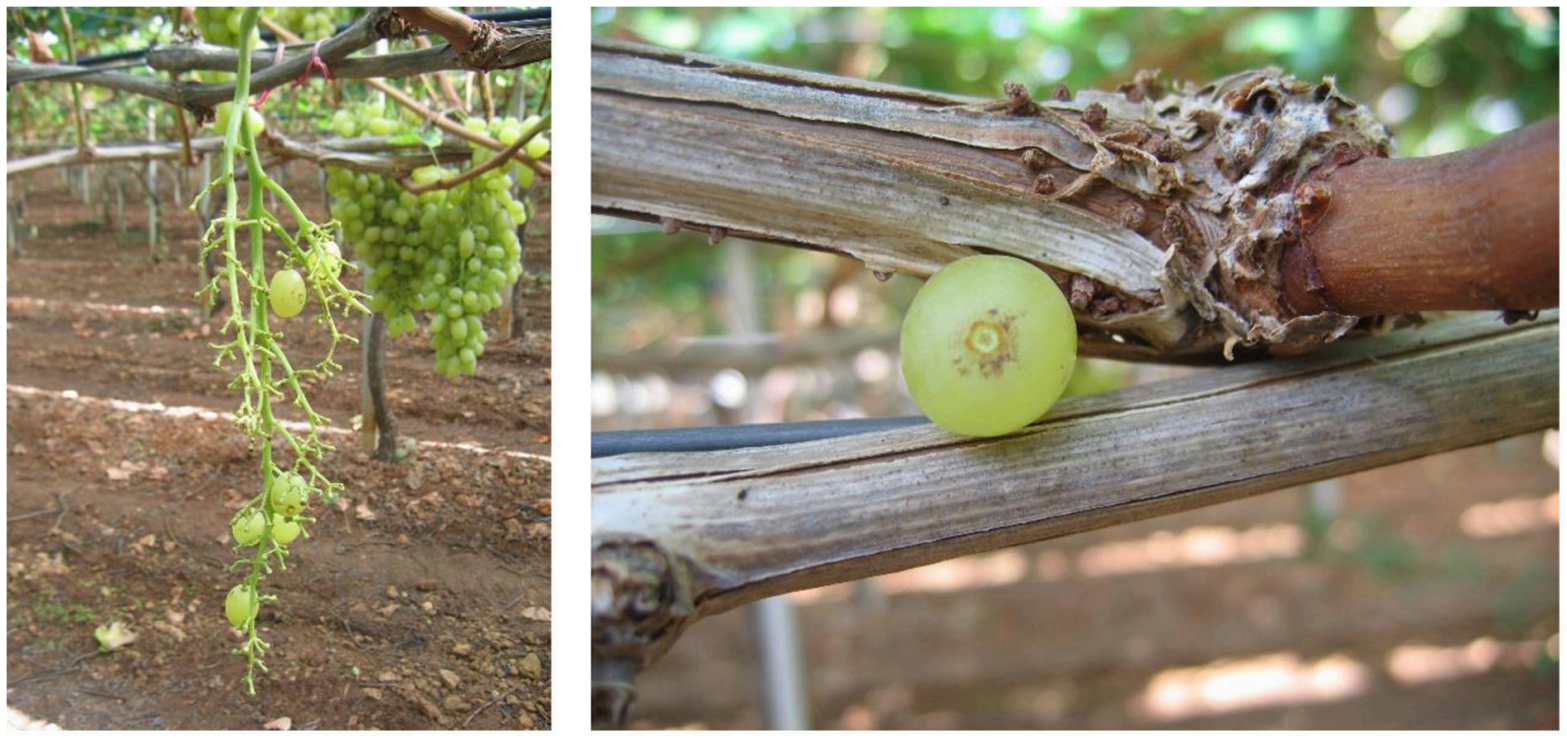
**Cluster of Thompson Seedless after the removal of the net to collect the abscised berries (left)**. Abscised berry with a dry stem scar **(right)**.

Ethephon treatments clearly reduced FDF because most of the berries on treated clusters were so loosely attached that they abscised before harvest or during handling (Figure 3). However, the few remaining berries on treated clusters were just as tightly held as the berries on non-treated clusters, so no treatment effects on FDF could be measured (Table 2). A similar result was reported for Thompson Seedless treated with methyl jasmonate, another abscission agent (González-Herranz et al., 2009). FDF may decline within a few days of treatment with abscission agents (El-Zeftawi, 1982; Uzquiza et al., 2013, 2015), so timely harvest may be needed when reductions in FDF are large. Abscission agents did not reduce fruit firmness, but FDF and berry firmness decreased from the time of ethephon application whether the clusters were treated or not (Table 2).

As suggested earlier, ethephon at either concentration tested stimulated an almost complete berry abscission from the rachis (Table 3). The effects of the two concentrations were similar, with only a few berries still attached to the rachis by harvest time (Figure 3), and the abscised berries generally had dry stem scars (Figure 3). Dry stem scars could be desirable for fresh-cut fruit since the scars help prevent juice leakage and minimize the exposure of interior berry tissues to the atmosphere and to pathogens that might reduce shelf-life or berry quality. However, pre-harvest berry abscission could lead to significant yield losses (Fidelibus et al., 2007), though yield loss might be minimized by earlier harvest or the use of catch systems, i.e., nets under the canopy.

**Table 3:** **Effects of different ethephon treatments on percentage of berry abscission, soluble solids content (SSC), pH and titratable acidity (TA) of Thompson seedless**.

		**Berry abscission (%)**	**SSC (%)**	**pH**	**TA (g/L)**
	**9 SEPTEMBER**				
Treatments	Control	0.0	19.5	3.66	5.7
	Eth 10^1^	0.0	19.0	3.61	5.6
	Eth 20^2^	0.0	19.0	3.58	5.5
	**21 SEPTEMBER**				
	Control	0.5	19.8	3.66	5.3
	Eth 10	94.3	19.6	3.64	5.7
	Eth 20	91.7	19.9	3.68	5.5
Time	**DATES**				
	9 Sept.	0.0^B^	19.2	3.62	5.6
	21 Sept.	62.2^A^	19.8	3.66	5.5

*Means followed by different letters in each column are significantly different (REGWQ, P ≤ 0.01).^1^Eth 10, ethephon at a concentration of 1445 mg/L; ^2^Eth 20, ethephon at a concentration of 2890 mg/L*.

Ethephon did not affect SSC, pH, or TA (Table 3). Few studies have examined the effect of abscission agents on grape berry composition, but our results generally agree with Uzquiza et al. (2015) who reported few and minor treatment effects on winegrapes. Even though a registered use of ethephon on grape is the promotion of fruit maturity, effects on grape composition are often variable, and ethephon applications to promote fruit maturity are made at veraison, a much earlier stage of fruit development (Szyjewicz et al., 1984). Abscission agents are applied to mature fruit, so there is less opportunity to affect fruit composition. Moreover, abscission agents quickly initiate the development of an abscission layer between the pedicel and berry (González-Herranz et al., 2009). The rapid action of abscission agents necessitates a short time period between application and harvest, further limiting the potential for differences in composition to develop.

### Physical and chemical analyses in Crimson seedless

Ethephon reduced the lightness (L^*^) and purity (C^*^) of the skin color (Table 4) as previously observed for Thompson Seedless, and similarly to that observed by others (Jayasena and Cameron, 2009). The FDF was significantly reduced (Table 4), whereas SSC and acidity were not affected as in a previous work (Jayasena and Cameron, 2009). A short post-harvest interval limits the possible compositional effects (El-Zeftawi, 1982), as discussed above. However, in a previous trial on Crimson Seedless, an increase of tartaric acid, procyanidin P2, terpenoid derivatives, and peonidin-3-glucoside as well as a decrease of catechin and epicatechin was observed after treatments with ethephon a few days before harvest (Rizzuti et al., 2015).

**Table 4:** Effects of different ethephon treatments on units of color space (L^*^, C^*^, and h°) of the berry skin, fruit detachment force (FDF) and firmness (whole berry and pulp) of Crimson seedless.

		L^*^	C^*^	**h°**	**FDF^3^**	**Berry^3^**	**Pulp^3^**
	**09 SEPTEMBER**						
Treatments	Control	25.9	5.0	8.1	9.0	4.29	0.67
	Eth 10^1^	26.5	5.1	7.8	8.9	4.19	0.68
	Eth 20^2^	25.9	5.2	8.8	9.1	4.03	0.67
	**21 SEPTEMBER**						
	Control	29.7^A^	9.4^A^	9.0	8.3^A^	4.05	0.54
	Eth 10	26.0^B^	7.9^AB^	4.9	6.2^B^	4.02	0.57
	Eth 20	26.9^B^	7.2^B^	4.7	6.3^B^	3.77	0.64
Time	**DATES**						
	9 Sept.	26.1^B^	5.1^B^	8.2	9.0^A^	4.17	0.67
	21 Sept.	27.5^A^	8.2^A^	6.2	6.9^B^	3.95	0.58

*Means followed by different letters in each column and for each date are significantly different (REGWQ, P ≤ 0.01). ^1^Eth 10, ethephon at a concentration of 1445 mg/L; ^2^Eth 20, ethephon at a concentration of 2890 mg/L; ^3^values in N*.

**Figure 4:**
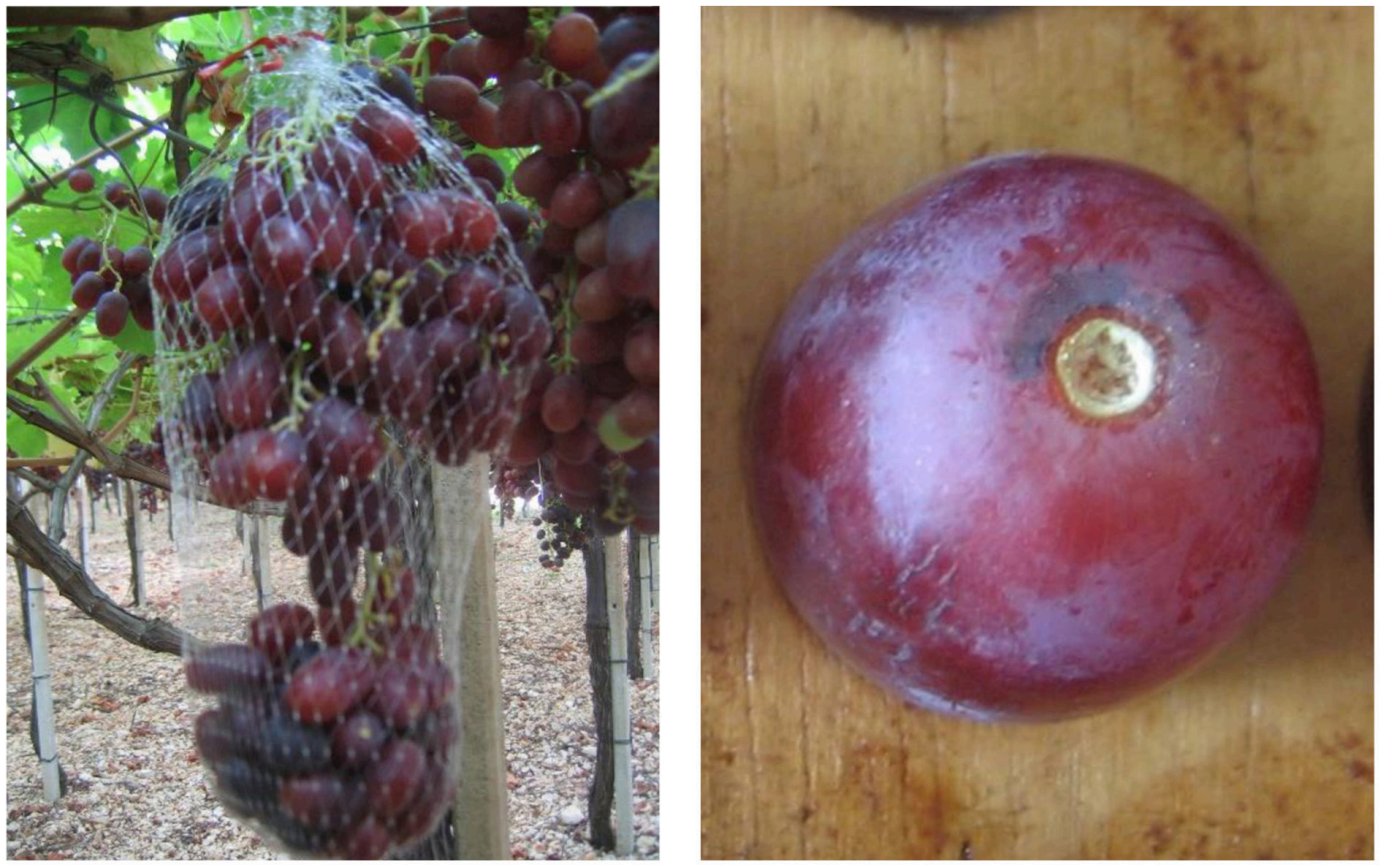
**Cluster of Crimson Seedless with abscised berries in the net (left)**. Abscised berry with a dry stem scar **(right)**.

Treatment with either concentration of ethephon stimulated significant pre-harvest abscission (Figure 4), both >40% and almost 55% at the dose of 2890 mg/L (Table 5). A similar effect on Crimson Seedless has been recently reported (Rizzuti et al., 2015). The treatments tested were less effective at inducing abscission of Crimson Seedless than they were at inducing abscission of Thompson Seedless. Differences among varieties in responsiveness to abscission agents has been previously reported in grape (Fidelibus et al., 2007), and it has also been observed that some table grape varieties are more susceptible than others to “shatter,” or “dry drop,” a post-harvest disorder characterized by the development of an abscission layer between the pedicel and berry (Lavee, 1959). The physiological basis for varietal differences in responsiveness to abscission agents is uncertain, but the application of very high rates of ethephon can induce abscission in varieties that are otherwise non-responsive (Fidelibus et al., 2007; Uzquiza et al., 2015), suggesting that the less responsive varieties may be less sensitive to ethylene. As observed with Thompson Seedless, SSC, pH, and TA of Crimson Seedless were not affected by abscission agents (Table 5). The lack of compositional effects are probably due to similar reasons identified and discussed earlier for Thompson Seedless.

**Table 5:** **Effects of different ethephon treatments on percentage of berry drop, soluble solids content (SSC), pH and titratable acidity (TA) of Crimson seedless**.

		**Berry abscission (%)**	**SSC (%)**	**pH**	**TA (g/L)**
Treatments	**09 SEPTEMBER**				
	Control	0.0	18.2	3.43	5.5
	Eth 10^1^	0.0	17.8	3.36	5.7
	Eth 20^2^	0.0	18.3	3.43	5.9
	**21 SEPTEMBER**				
	Control	0.0^B^	18.9	3.52	5.3
	Eth 10	44.0^A^	18.5	3.44	5.6
	Eth 20	54.9^A^	18.6	3.50	5.3
Time	**DATES**				
	9 Sept.	0.0^B^	18.1	3.41	5.7
	21 Sept.	33.0^A^	18.7	3.49	5.4

*Means followed by different letters in each column and for each date are significantly different (REGWQ, P ≤ 0.01). ^1^Eth 10, ethephon at a concentration of 1445 mg/L; ^2^Eth 20, ethephon at a concentration of 2890 mg/L*.

### Ethephon residues in the berry

**Figure 5:**
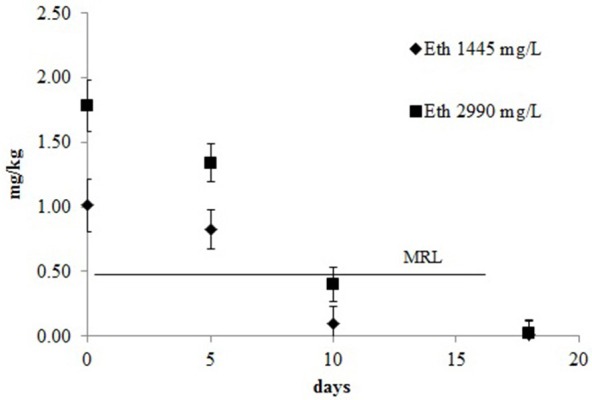
**Decay kinetic model of ethephon for table grape**.

The decay kinetics model of ethephon is shown in Figure 5. The proposed model was a single first order kinetic. When the concentration of ethephon used was 1445 mg/L the *R*^2^ value of the model was 0.84 and the DT50 e DT90 were 5.3 and 17.6 days, respectively. With the higher concentration the kinetic model had a better fit (*R*^2^ = 0.91) and slightly higher DT50 and DT90 at the values of 5.7 and 18.9, respectively. As expected, the residual concentration declined rapidly until harvest, reaching levels below the MRL 5 or 6 days after treatment. The recommended pre-harvest interval of 16 days from the treatment is well defined and lead to a level of residues in the grape clearly below the MRL regardless of dose.

[235pt]

## Conclusion

The use of ethephon on Thompson Seedless resulted in almost complete berry abscission by harvest, whereas its efficacy was more limited for Crimson Seedless. Berries of both varieties did not show pronounced changes in visual, physical or chemical properties that could interfere with a possible use as fresh-cut fruit (Supplementary Image 1), and they also presented a desirable dry stem scar. Residues were generally below the limits, at least for the lower concentration tested. Our data suggested the application of 1445 mg/L ethephon to Thompson Seedless is sufficient to reduce FDF and promote the development of dry stem scars without resulting in residues that exceed current MRL for ethephon.

## Author contributions

All authors listed, have made substantial, direct and intellectual contribution to the work, and approved it for publication.

### Conflict of interest statement

The reviewer CHC declared a shared affiliation, though no other collaboration, with one of the authors MWF to the handling Editor, who ensured that the process nevertheless met the standards of a fair and objective review. The other authors declare that the research was conducted in the absence of any commercial or financial relationships that could be construed as a potential conflict of interest.
