# Supplementary figures and images for: Ethephon As a Potential Abscission Agent for Table Grapes: Effects on Pre-Harvest Abscission, Fruit Quality, and Residue
Source: Front Plant Sci. 2016 May 30;7:620. doi: 10.3389/fpls.2016.00620 (PMC4885227; doi:10.3389/fpls.2016.00620)

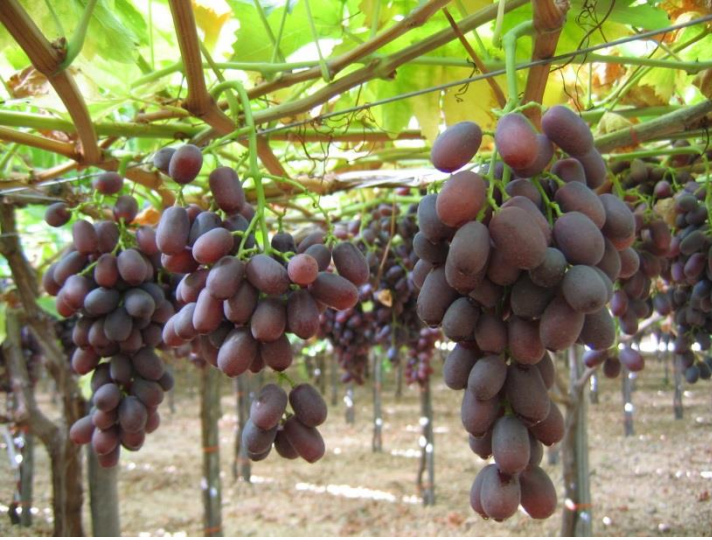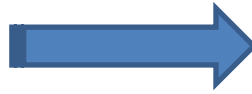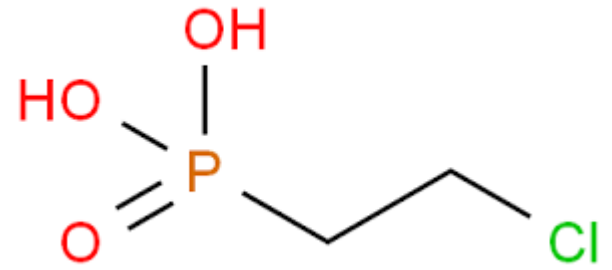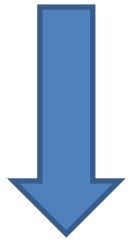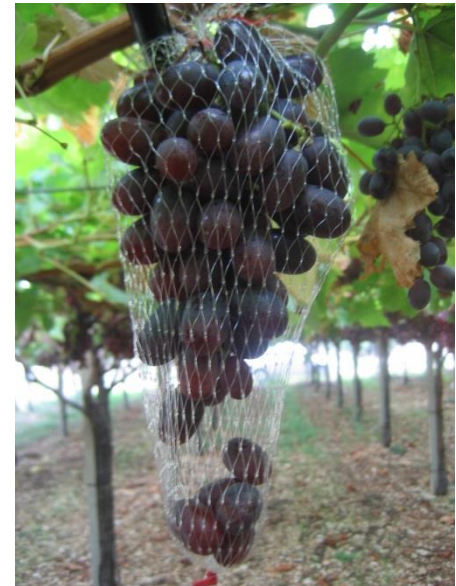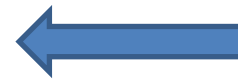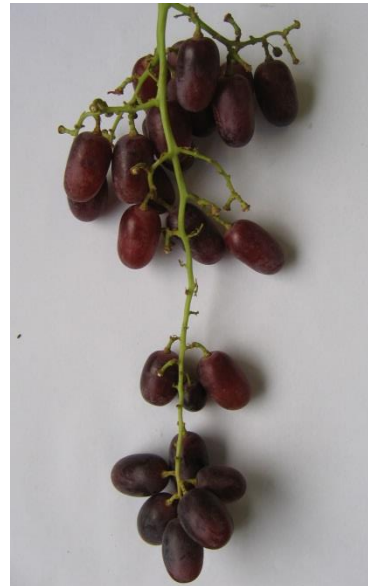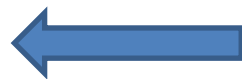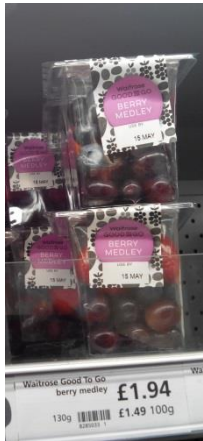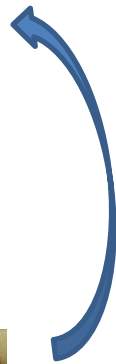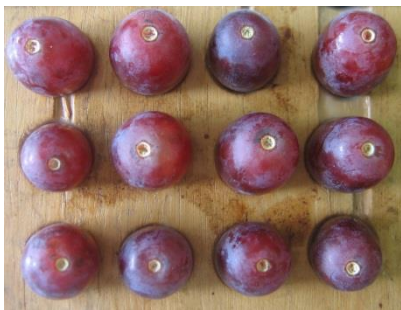

Supplement: Supplementary file 1 [file Image1.PDF]
